# Aldehyde Dehydrogenase 1 making molecular inroads into the differential vulnerability of nigrostriatal dopaminergic neuron subtypes in Parkinson’s disease

**DOI:** 10.1186/2047-9158-3-27

**Published:** 2014-12-10

**Authors:** Huaibin Cai, Guoxiang Liu, Lixin Sun, Jinhui Ding

**Affiliations:** Transgenics Section, Laboratory of Neurogenetics, National Institute on Aging, National Institutes of Health, Bethesda, MD 20892 USA; Computational Biology Core, Laboratory of Neurogenetics, National Institute on Aging, National Institutes of Health, Bethesda, MD 20892 USA

**Keywords:** Parkinson’s disease, *Substantia nigra pars compacta*, Dopaminergic neuron, Aldehyde dehydrogenase 1, α-synuclein, Neurodegeneration, Aging

## Abstract

A preferential dysfunction/loss of dopaminergic (DA) neurons in the *substantia nigra pars compacta* (SNpc) accounts for the main motor symptoms of Parkinson’s disease (PD), the most common degenerative movement disorder. However, the neuronal loss is not stochastic, but rather displays regionally selectivity, indicating the existence of different DA subpopulations in the SNpc. To identify the underlying molecular determinants is thereby instrumental in understanding the pathophysiological mechanisms of PD-related neuron dysfunction/loss and offering new therapeutic targets. Recently, we have demonstrated that aldehyde dehydrogenase 1 (ALDH1A1) is one such molecular determinant that defines and protects an SNpc DA neuron subpopulation preferentially affected in PD. In this review, we provide further analysis and discussion on the roles of ALDH1A1 in the function and survival of SNpc DA neurons in both rodent and human brains. We also explore the feasibility of ALDH1A1 as a potential biomarker and therapeutic target for PD.

## Introduction

Parkinson’s disease (PD), clinically manifested with resting tremor, dyskinesia/akinesia, posture instability, rigidity, and other motor symptoms [1], results from a preferential dysfunction/loss of the *substantia nigra pars compacta* (SNpc) dopaminergic (DA) neurons [2]. As supporting evidence, dopamine replacement therapy using the dopamine precursor L-3, 4-dihydroxyphenylalanine (L-DOPA) has been broadly employed to alleviate the motor symptoms [3]. Although L-DOPA is the gold standard PD therapy, it cannot prevent the progressive loss of SNpc neurons and becomes less effective at the late stages of the disease [4]. In addition, some patients respond poorly to the administration of L-DOPA, while others develop dyskinesia [5]. To understand why the SNpc DA neurons are preferentially susceptible to degeneration and how to prevent it remain the most challenging questions in PD research and treatment. Here we discuss recent advances in the identification of key molecular determinants critical for the survival of a subpopulation of SNpc DA neurons selectively degenerated in PD.

## SNpc regional selectivity in aging and PD

SNpc DA neurons are highly specialized and possess many distinct morphological and functional properties [6]. They have long, unmyelinated and highly ramified axons [7]; use highly reactive dopamine as the transmitter [8]; and, function as a pace-maker using calcium currents [6, 9]. SNpc DA neurons likely undertake tremendous stress to support their constant neural activities, to dispose cytotoxic dopamine metabolites, and to maintain calcium homeostasis, which likely render them more susceptible to aging, PD, and other risk factors [6, 9, 10].

Despite sharing many distinct features as mentioned above, SNpc DA neurons are not a homogeneous population of neurons [11–13]. They seem to organize into different subdivisions within the SNpc and display differential vulnerability during aging and PD processes [11–13]. Based on the pattern of neuronal loss in normal aging and PD, Fearnley and Lees divide human SNpc into six morphometric regions, including the medial part (DM), lateral part (DL) and pars lateralis (PL) in the dorsal tier, and the medial part (VM), intermediate part (VI) and lateral part (VL) in the ventral tier [12]. It appears that normal aging mainly affects the DA neurons distributed in the dorsal tier of SNpc, whereas PD causes additional and more severe loss of DA neurons in the ventral tier, especially the VL subpopulation [12]. Fearnley and Lees further postulate that SNpc DA neurons undergo a biphasic mode of degeneration in PD comprised of an age-dependent linear phase and a PD-induced accelerated phase of neuronal loss [12]. These earlier anatomical observations imply the existence of distinct molecular determinants that define and protect SNpc subpopulations selectively affected in PD. To identify the underlying molecular clues may not only shed light on the cause of SNpc DA neuronal loss in PD, but also provide new biomarkers and therapeutic targets for the treatment of the disease.

## ALDH1A1 defines a subpopulation of SNpc DA neurons in both rodent and human brains

**Figure 1:**
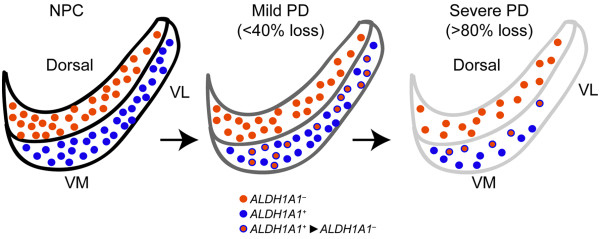
**Selective loss of ALDH1A1-positive subtype nigrostriatal DA neurons in PD.** Cartoons illustrate the distribution of ALDH1A1–negative (ALDH1A1^−^, red) and –positive (ALDH1A1^+^, blue) DA neurons, as well as ALDH1A1^+^ neurons that lose ALDH1A1 expression (ALDH1A1^+^➔ ALDH1A1^−^, red with blue outline) in the SNpc of non-pathology control (NPC) and PD brains with mild and severe depigmentation. VL: ventral lateral, VM: ventral medial.

**Figure 2:**
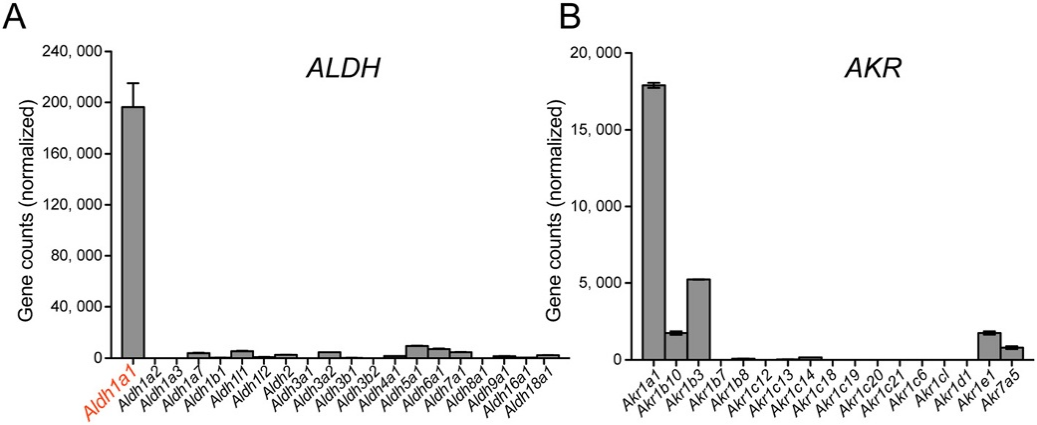
**Expression of** ***Aldh*** **and** ***Akr*** **family genes in the mouse SNpc DA neurons. (A, B)** RNA sequencing reveals the expression of *Aldh* **(A)** and *Akt* **(B)** family genes in SNpc DA neurons of 12-month-old wild type mice. Two independent sets of SNpc RNA samples were analyzed.

SNpc DA neurons express a selective set of genes encoding proteins critical for the synthesis, transport and degradation of dopamine, including tyrosine hydroxylase (TH), vesicular monoamine transporter 2 (VMAT2), dopamine transporter (DAT), and aldehyde dehydrogenase 1 (ALDH1A1) [14]. In contrast to a ubiquitous expression pattern of TH, VMAT2, and DAT in all SNpc DA neurons, ALDH1A1 appears to be expressed only in DA neurons residing at the ventral tier of rodent SNpc [15]. Moreover, a conserved topographic distribution of ALDH1A1-positive SNpc DA neurons is also observed in the ventral tier of human SNpc (Figure 1) [16]. Therefore, based on the expression of ALDH1A1, SNpc DA neurons can be divided into two subtypes that exhibit different susceptibility in PD (Figure 1) [16]. ALDH1A1 belongs to a large family of ALDH genes that consist of 19 members in the human genome [17]. Interestingly, among all ALDH genes only *Aldh1a1* is predominantly and highly expressed by the SNpc DA neurons in the mouse CNS (Figure 2A) [16, 18]. The expression of *Aldh1a1* mRNA was also much higher than any aldehyde reductase (AKR) genes, which may also involve with the oxidization of dopamine-3, 4-dihydroxyphenylacetaldehyde (DOPAL) (Figure 2B) [19]. Hence ALDH1A1 may possess some distinct characteristics critical for the function and survival of a subset of SNpc DA neurons preferentially degenerated in PD. Meanwhile, it is perhaps more accurate to pathologically characterize PD as caused by a selective loss of ALDH1A1-positive SNpc DA neurons.

## ALDH1A1 functions in periphery tissues and SNpc DA neurons

ALDH1A1 proteins exist as homotetramers in the cytosol, and oxidize various cytosolic aldehyde intermediates into the chemically more inert acidic forms [20]. The oxidative activity of ALDH1A1 requires the presence of the co-factor oxidized nicotinamide adenine dinucleotide (NAD^+^) [17]. ALDH1A1 has been involved in the metabolism of alcohol in the liver and retinol in the eye, brain, and other tissues [21]. ALDH1A1-deficiency has been indicated in alcohol-intolerance and cornea opacity [17]. On the other hand, an abnormal increase of ALDH1A1 has been observed in certain cancers, while ALDH1A1 inhibitors have been developed for cancer therapy [17, 22].

In the brain, ALDH1A1-mediated production of retinoid acid (RA) is required for the differentiation of DA neurons during embryonic development [23]. The expression of *Aldh1a1* in the midbrain DA neurons is under the transcriptional control of pituitary homeobox 3 (*Pitx3*) and forkhead box protein A1/2 (*Foxa1/2*) [23, 24]. A lack of *Pitx3* impairs the expression of *Aldh1a1* and the terminal differentiation of midbrain DA neurons, whereas a supplement of RA in embryos rescues the developmental defects caused by *Pitx3*-deficiency [23]. Since *Pitx3* and *Foxa1/2* show rather ubiquitous expression pattern in the midbrain DA neurons, they may not be responsible for the selective expression of *Aldh1a1* in the SNpc. It would be interesting to identify additional upstream transcription factors that regulate the expression of *Aldh1a1* selectively in a subpopulation of DA neurons.

**Figure 3:**
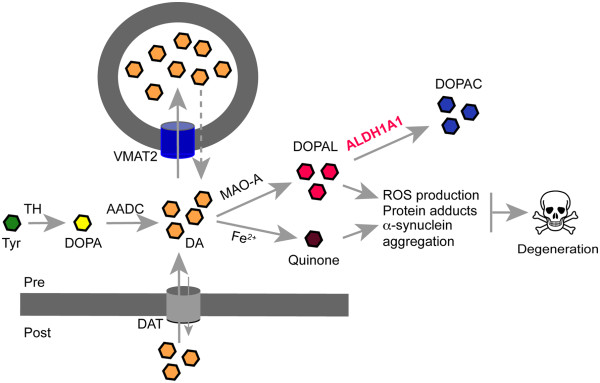
**Dopamine metabolism and selective loss of SNpc DA neurons.** Cartoon proposes the accumulation of cytosolic DOPAL triggers the degeneration of SNpc DA neurons in PD. Tyrosine hydroxylase (TH) and aromatic L-amino acid decarboxylase (AADC) mediate the synthesis of dopamine from tyrosine (Tyr) in the cytosol. Cytosolic dopamine is then immediately sequestered into the synaptic vesicles (SVs) by dopamine transporter VMAT2 for release. DAT mediates the reuptake of dopamine from extracellular space into the DA terminals. Leakage of dopamine from SVs also contributes to the cytosolic dopamine levels. MAO and ALDH1A1 are main enzymes for the degradation of cytosolic dopamine in DA neurons. A lack of ALDH1A1 may lead to a cytotoxic build-up of DOPAL which triggers the reactive oxygen species (ROS) production, protein adducts and α-synuclein aggregation, and eventually leads to cell death.

Additionally, ALDH1A1 also mediates the oxidation of DOPAL in DA neurons (Figure 3) [20]. Dopamine is produced in the cytosol before being sequestrated into the synaptic vesicles by VMAT2 (Figure 3) [25]. On the other hand, vesicular dopamine seems to constantly leak into the cytosol (Figure 3) [26]. Free cytosolic dopamine may undergo autoxidation to form cytotoxic quinones and other free radicals (Figure 3) [27]. To remove the cytosolic dopamine and its byproducts, monoamine oxidases (MAOs), ALDHs and AKRs are employed to degrade cytosolic dopamine and DOPAL into 3, 4-Dihydroxyphenylacetic acid (DOPAC) and other less reactive metabolites (Figure 3) [20, 26]. DOPAL is highly reactive and a lack of ALDH1A1 may lead to accumulation of DOPAL that has been shown to promote cytotoxic polymerization of PD-related α-synuclein and compromise the functions of proteins important in the activity and survival of SNpc DA neurons (Figure 3) [28]. In support of this notion, exposure of fungicide benomyl, an inhibitor of aldehyde dehydrogenase increases the risk of PD [29].

Given the importance of ALDH1A1 in dopamine metabolism, why ALDH1A1 is only expressed by a subset of DA neurons remains an intriguing question. In the absence of ALDH1A1, other ALDH and AKRs family proteins likely substitute its role in the oxidation of DOPAL and other cytosolic aldehyde intermediates. However, except for ALDH1A1, no other ALDHs or AKRs are particularly enriched in the SNpc DA neurons, or restricted to any subpopulations (Figure 2A, B) [16] (Allen Brain Atlas). We speculate the highly selective expression of ALDH1A1 in the ventral subpopulation of SNpc DA neurons may provide extra protection for these neurons that are preferentially vulnerable in PD [12].

## ALDH1A1 contributes to the preferential loss of ventral SNpc DA neurons in PD

PD brains are featured with a more severe loss of ventral SNpc DA neurons [12]. One of the common molecular properties of these neurons is the expression of ALDH1A1 [16]. Correlatively, a more severe loss of ventral ALDH1A1-positive SNpc DA neurons has been observed in the PD cases (Figure 1) [16]. More interestingly, a significant increase of ALDH1A1-negative DA neurons in the ventral tier of SNpc is observed in the mild PD cases compared to the normal controls (Figure 1) [16]. A likely explanation of this observation is that PD may initially cause a reduction of ALDH1A1 expression in the ALDH1A1-positive DA neurons prior to the eventual neuronal loss. Reductions of *ALDH1A1* mRNA and protein expression have also been reported in the SNpc of postmortem PD brains [30–32]. Moreover, in the α-synuclein transgenic mice both *Aldh1a1* mRNA and protein levels are also decreased in DA neurons [16]. These findings suggest that ALDH1A1 itself is also a pathogenic target in PD. The reduction of ALDH1A1 expression in PD may weaken the protective function of ALDH1A1 in the ventral tier of SNpc, and predispose these neurons to degeneration at the later stages of disease. Therefore, a severe loss of ALDH1A1 expression may represent the turning point for ventral SNpc DA neurons that degenerate in PD. The expression level and activity of ALDH1A1 may serve as a useful biomarker to monitor the progression of the disease.

## Mouse ALDH1A1-positive SNpc DA neurons are more resistant to α-synuclein-induced neurodegeneration

α-synuclein is a prominent genetic causal factor in the pathogenesis of PD [33–37]. A potential pathogenic interaction between cytosolic dopamine and α-synuclein has been implicated in the pathogenesis of PD [27, 38]. One of the key pathogenic mechanisms of α-synuclein in DA neurons is to form cytotoxic protein aggregates that may impair the synthesis, uptake, and degradation of dopamine [39–41]. The increased formation of cytotoxic dopamine quinones and DOPAL, on the other hand, may further promote α-synuclein aggregation through polymerization of monomeric α-synuclein [42, 43]. This pathogenic interplay between reactive dopamine derivatives and α-synuclein aggregation may form a vicious cycle that amplifies their detrimental effects to the DA neurons [42].

When the PD-related α-synuclein A53T missense mutation is introduced into the midbrain DA neurons, the resulting *Pitx3–tTA::tetO–A53T* bigenic mice develop profound motor disabilities and robust SNpc DA neuron loss [41]. Interestingly, the degenerated neurons are mainly distributed at the dorsal medial tier of SNpc that lack ALDH1A1 expression [16]. Noticeably, more cytotoxic α-synuclein aggregates are present in ALDH1A1-negative population of SNpc DA neurons in the mutant mice [16], suggesting that more DOPAL or other reactive dopamine intermediates may be present in these neurons to promote α-synuclein polymerization and aggregation [42, 43]. By contrast, the ventral ALDH1A1-postive SNpc DA neurons contain less α-synuclein aggregates and appear to resist α-synuclein-induced neuron loss during the aging process [16], thereby supporting the protective role of ALDH1A1 in these neurons. Correlatively, genetic deletion of *Aldh1a1* gene exacerbates α-synuclein-induced SNpc DA neuronal loss in the *Aldh1a1* knockout mice [16]. It is necessary to point out that the subtypes of the remaining SNpc DA neurons were not defined in the *Aldh1a1*-deficient mice due to a lack of molecular markers. Future studies will be required to identify additional molecular markers for different subtypes of SNpc DA neurons. To directly support the protective function of ALDH1A1 for DA neurons, over-expression of ALDH1A1 selectively ameliorates α-synuclein-induced cytotoxicity in the cultured DA neurons [16]. It will be interesting to examine the protective role of ALDH1A1 *in vivo* through the overexpression of ALDH1A1 or the use of selective activators. This proposed study may pave the way for establishing ALDH1A1 as an important therapeutic target for PD.

## ALDH1A7 is highly homologous to ALDH1A1 and only exists in mouse but not human genome

There still lacks a pathologically more accurate PD mouse model that shows progressive loss of ALDH1A1-positive SNpc DA neurons. Why the mouse ALDH1A1-postive SNpc DA neurons are more resistant to α-synuclein-induced degeneration remains speculative [16]. Although ALDH1A1 appears to play an important role in the development and maintenance of SNpc DA neurons [16, 23], genetic deletion of *Aldh1a1* fails to produce any overt motor symptoms or SNpc DA neuron loss in the *Aldh1a1* knockout mice [44, 45]. Other ALDHs or AKRs may compensate for the loss of ALDH1A1 as shown in the *Aldh1a1* and *Aldh2* double knockout mice that develop mild but statistically significant loss of SNpc DA neurons [45].

**Figure 4:**
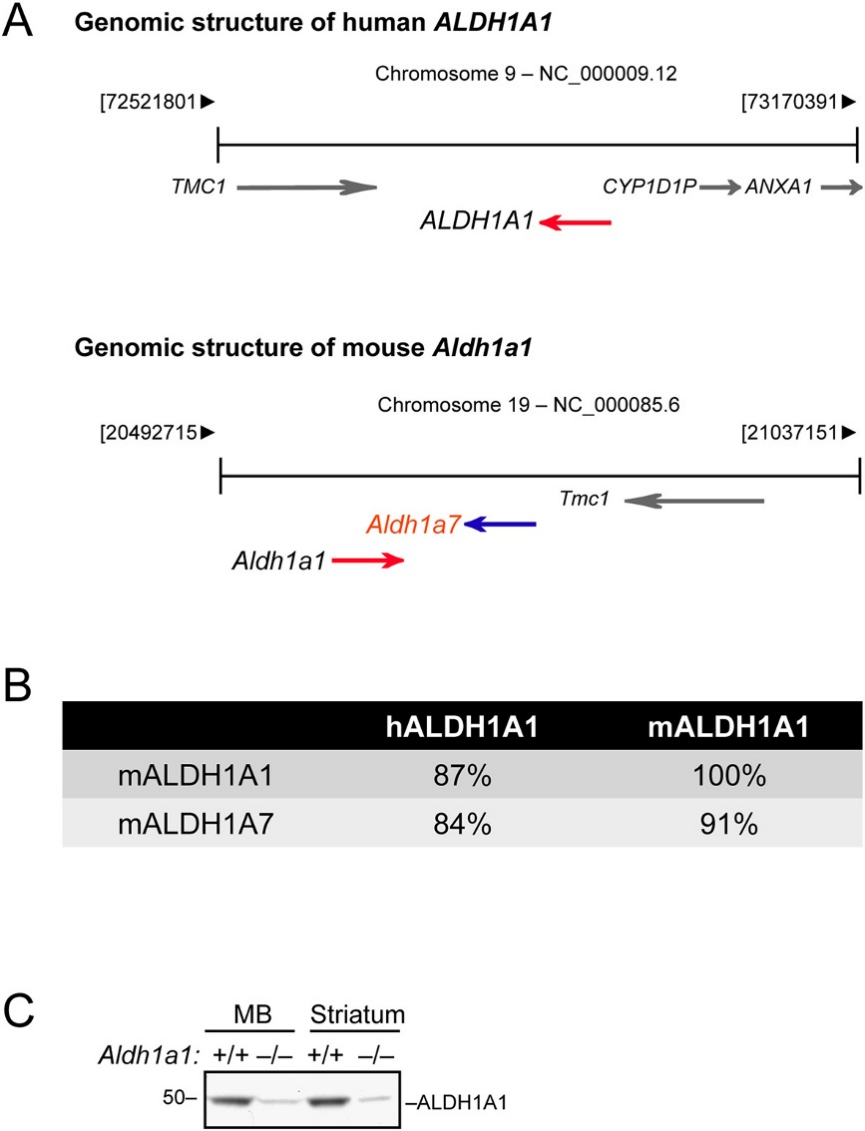
**ALDH1A7 is highly homologous to ALDH1A1 in the mouse genome. (A)** Diagrams depict the genomic structures of human *ALDH1A1* (*hALDH1A1*) and mouse *Aldh1a1* (*mAldh1a1*) and *Aldh1a7* (*mAldh1a7*). Arrows point to the direction of transcription. **(B)** Table shows the percentage of identical amino acids shared between hALDH1A1, mALDH1A1, and mALDH1A7 proteins. **(C)** Western blot shows the residual proteins recognized by an ALDH1A1 antibody in both the midbrain (MB) and striatum of *Aldh1a1* ^–/–^ mice.

Interestingly, when comparing the genomic organization of mouse and human *ALDH1A1*, mouse *Aldh1a1* sits side-by-side with *Aldh1a7* in chromosome 19, whereas no corresponding *ALDH1A7* is found in the human genome (Figure 4A). Mouse ALDH1A7 proteins share 91% and 84% identical residues with mouse and human ALDH1A1, respectively, suggesting that ALDH1A1 and ALDH1A7 may have interchangeable functionalities (Figure 4B). The mouse *Aldh1a1* and *Aldh1a7* genes are likely derived from an ancestor gene through gene duplication, an event that seems not evolutionally conserved between mice and humans. In the midbrain lysates of *Aldh1a1* homozygous knockout mice, around 80% reduction of ALDH1A1 protein expression was detected (Figure 4C). The remaining 20% of proteins likely reflect the expression of ALDH1A7, since the ALDH1A1 antibody possibly also recognizes ALDH1A7 due to the high homology shared by these two proteins. The presence of ALDH1A7 may thereby provide extra protection for the SNpc DA neurons in the mouse brains. Why mice but not human have ALDH1A7 is a mystery. However, ALDH1A7 may provide extra protection to the SNpc DA neurons in mouse brains.

## ALDH1A1 as a biomarker and therapeutic target in PD

ALDH1A1 may exert its protective function to SNpc DA neurons via mitigating the cytotoxicity of DOPAL. A substantial reduction of ALDH1A1 expression and severe loss of ALDH1A1-positive SNpc DA have been observed in the postmortem PD brains [16, 30]. Collaboratively, studies of postmortem brains show a drop of DOPAC content in the putamen of PD patients, reflecting the reduced ALDH1A1 activity [14]. In addition, gene expression profiling of whole blood samples from 105 PD patients shows that *ALDH1A1* mRNA together with other three genes are specific indicators for PD diagnosis since no such changes are found in control as well as Alzheimer’s cases [46]. Although the transcriptional regulation of *ALDH1A1* may differ in SNpc DA neurons and blood cells, a similar systematic alteration of its expression might occur in both the CNS and periphery tissues. The levels of *ALDH1A1* expression and activity either in the blood or CSF could serve as biomarkers for the diagnosis of PD.

The reduction of ALDH1A1 expression in PD might be employed as a compensatory mechanism to boost the release of dopamine in the remaining SNpc DA neurons via slowing down the turnover of dopamine. However, the undesired consequence of this approach is the resulting DOPAL-induced cytotoxicity, such as increased oxidative stress, protein adducts, and α-synuclein aggregation [47]. ALDH1A1 activation could be applied to suppress the toxic effects of DOPAL in the PD brains. Previous studies in cancer research have identified a number of intracellular signaling pathways that lead to an increase expression of ALDH1A1 in cancer cells [17]. However, whether SNpc DA neurons adopt the same pathways in regulating *ALDH1A1* mRNA expression remains to be determined. In addition, it would be important to evaluate the impact of posttranslational modifications on the expression and function of ALDH1A1 proteins. The knowledge gained from these studies may help to design potential therapeutic interventions that boost the activity of ALDH1A1 in the PD brains.

On the other side, a variety of ALDH inhibitors have been developed to treat cancers, alcohol abuse and other disorders [17]. Among them, disulfiram, an alcohol-aversive drug [48], exhibits more potent inhibition of ALDH1A1 than ALDH2 and other ALDHs [49]. However, whether the administration of ALDH1A1 inhibitors may increase the risk of PD remains to determine. In addition, it would be interesting to learn if the experience obtained from designing ALDH1A1 inhibitors would help to produce ALDH1A1 specific activators for the treatment of PD.

## Conclusions

Increasing evidence points out the significance of cytotoxic DOPAL and other dopamine metabolites in causing PD-related DA neurodegeneration [47]. ALDH1A1 is a key enzyme that irreversibly oxidizes DOPAL into less toxic DOPAC [20]. The presence of ALDH1A1 in a subpopulation of SNpc DA neurons not only defines a neuronal subtype selectively susceptible in PD, but also opens a new window to further characterize the connectivity and functionality of this important group of neurons in dopaminergic transmission [16]. Therefore, elucidation of the molecular and pathophysiological properties of these ALDH1A1-positive SNpc DA neurons may provide major advancement on our current understanding of the pathogenic mechanism of PD-related neuronal loss and lead to better treatment of the disease.
